# Choosing the Right Vasoactive Agent in Acute Variceal Bleeding in Cirrhotic Patients: A Review of Terlipressin vs. Octreotide Outcomes

**DOI:** 10.7759/cureus.87910

**Published:** 2025-07-14

**Authors:** Moses John Wesley, Anjali Avula, Hafsa Tehniyat, Muhammad Hamza Zamir, Umer Farooq, Fizza Tahir, Almas Akbar, Kirshan Lal, Katherine S Trejos Guzman, Muhammad M Tariq, Muhammad Samaat, Abdul Haseeb Hasan

**Affiliations:** 1 Internal Medicine, Annai Hospital, Thuraiyur, IND; 2 Internal Medicine, Guntur Medical College, Guntur, IND; 3 Internal Medicine, Medicore Clinic, Hyderabad, IND; 4 Internal Medicine, Fauji Foundation Hospital, Rawalpindi, PAK; 5 Internal Medicine, Rawalpindi Medical University, Rawalpindi, PAK; 6 Pediatrics, Shaheed Mohtarma Benazir Bhutto Medical University, Larkana, PAK; 7 Internal Medicine, National Autonomous University of Nicaragua, Managua, NIC; 8 Internal Medicine, Foundation University Medical College, Islamabad, PAK; 9 Internal Medicine, Mayo Hospital, Lahore, PAK

**Keywords:** acute variceal bleeding, cirrhosis, hemodynamics, octreotide, portal hypertension, randomized controlled trials, systematic review, terlipressin

## Abstract

This systematic review evaluates and compares the clinical effectiveness of terlipressin and octreotide in the management of acute gastroesophageal variceal bleeding among cirrhotic patients. A comprehensive literature search was conducted across PubMed, Google Scholar, and clinical trial registries, identifying five randomized controlled trials that met predefined inclusion criteria. These studies assessed key outcomes, including bleeding control, mortality, hepatic venous pressure gradient (HVPG) reduction, hemodynamic stability, and safety profiles. The findings demonstrated that both agents are effective in controlling variceal bleeding, with no major differences in short-term mortality. However, terlipressin was associated with sustained HVPG reduction, more durable hemodynamic effects, and, in some cases, shorter hospital stays. In alcoholic cirrhosis subgroups, terlipressin showed a superior portal pressure response. Risk of bias assessments indicated generally low-to-moderate concerns across studies, supporting the reliability of findings. This review highlights terlipressin’s potential advantages in specific clinical contexts and underscores the need for further large-scale trials to refine therapy selection and optimize patient outcomes.

## Introduction and background

Acute variceal bleeding (AVB) remains one of the most life-threatening complications in patients with liver cirrhosis, accounting for significant morbidity and mortality worldwide [1]. Gastroesophageal varices develop in response to increased portal hypertension and are present in over half of cirrhotic patients. When bleeding occurs, immediate intervention is critical, with initial management focusing on hemodynamic stabilization, pharmacological vasoconstriction, and endoscopic therapy [2]. Despite advances in care, mortality rates associated with AVB still hover around 15-20%, especially in decompensated liver disease [3].

Among pharmacologic therapies, vasoactive agents such as terlipressin and octreotide have played a central role. Terlipressin, a vasopressin analog, exerts potent splanchnic vasoconstrictive effects with a longer half-life and reduced risk of systemic side effects [4]. Octreotide, a somatostatin analog, inhibits glucagon-induced splanchnic vasodilation and decreases portal venous inflow [5]. Both agents are typically used in conjunction with endoscopic band ligation as part of the standard of care, but the debate remains regarding their comparative efficacy in controlling bleeding, reducing early rebleeding, and improving short-term survival [6]. Additionally, their varying hemodynamic profiles and side-effect spectra raise important clinical considerations in individualized patient management.

The objective of this systematic review is to evaluate and compare the clinical effectiveness of terlipressin versus octreotide in managing AVB in adult patients with liver cirrhosis, focusing on key outcomes, such as bleeding control, rebleeding rates, mortality, and adverse events. By synthesizing data from relevant randomized controlled trials, this study aims to inform clinicians about the most effective pharmacological option in the acute management of variceal hemorrhage.

## Review

Materials and methods

Study Design and Framework

This study was conducted as a systematic review in accordance with the Preferred Reporting Items for Systematic Reviews and Meta-Analyses (PRISMA) guidelines [7]. The objective was to compare the efficacy and clinical outcomes of terlipressin versus octreotide in the management of acute variceal bleeding in patients with cirrhosis. The review adhered to a structured PICO (population, intervention, comparison, and outcome) framework [8]. The population included adult patients with cirrhosis presenting with gastroesophageal variceal hemorrhage; intervention was terlipressin therapy; the comparator was octreotide therapy; and the outcomes assessed included bleeding control, mortality, hepatic venous pressure gradient (HVPG), and hemodynamic parameters.

Search Strategy

A comprehensive literature search was conducted using PubMed, Google Scholar, and clinical trial registries to identify relevant randomized controlled trials (RCTs) published in English. The search included a combination of terms such as “terlipressin”, “octreotide”, “variceal bleeding”, “esophageal varices”, “portal hypertension”, and “randomized controlled trial”. Boolean operators and truncations were used as appropriate. The search was supplemented by manually reviewing the reference lists of key articles to identify additional relevant studies. The search encompassed studies up to April 2025.

Eligibility Criteria

Studies were selected based on pre-defined inclusion criteria: (1) RCTs comparing terlipressin and octreotide in adult cirrhotic patients with acute gastroesophageal variceal bleeding; (2) studies reporting on at least one clinical outcome of interest, such as bleeding control, mortality, HVPG reduction, or safety profile; and (3) articles published in English with full-text availability. Exclusion criteria included non-randomized designs, pediatric populations, non-cirrhotic etiologies, and lack of comparative outcome data.

Study Selection and Data Extraction

All identified articles were screened independently by two reviewers. Initial screening was based on titles and abstracts, followed by full-text review of eligible studies. Discrepancies were resolved through discussion and consensus. Key data extracted from each study included the first author’s name, year of publication, study design, sample size, patient population, intervention and comparator details, and primary clinical outcomes. Extracted data were compiled in a comparative table for systematic qualitative analysis.

Risk of Bias Assessment

The methodological quality of included studies was evaluated using the Cochrane Risk of Bias (RoB 2.0) tool [9]. This involved assessing domains such as randomization process, allocation concealment, blinding, completeness of outcome data, and selective reporting. Each study was categorized as having low risk, some concerns, or high risk of bias. The results were summarized in a tabulated form to enhance the transparency and reliability of the evidence presented.

Data Synthesis and Analysis

Due to clinical and methodological heterogeneity among the included trials - such as differences in drug dosage, timing of administration, and outcome definitions - a narrative synthesis was conducted rather than a statistical meta-analysis. Trends in bleeding control, hemodynamic responses, and patient-centered outcomes were compared qualitatively across the studies to derive clinically meaningful interpretations.

Results

Study Selection Process

**Figure 1:**
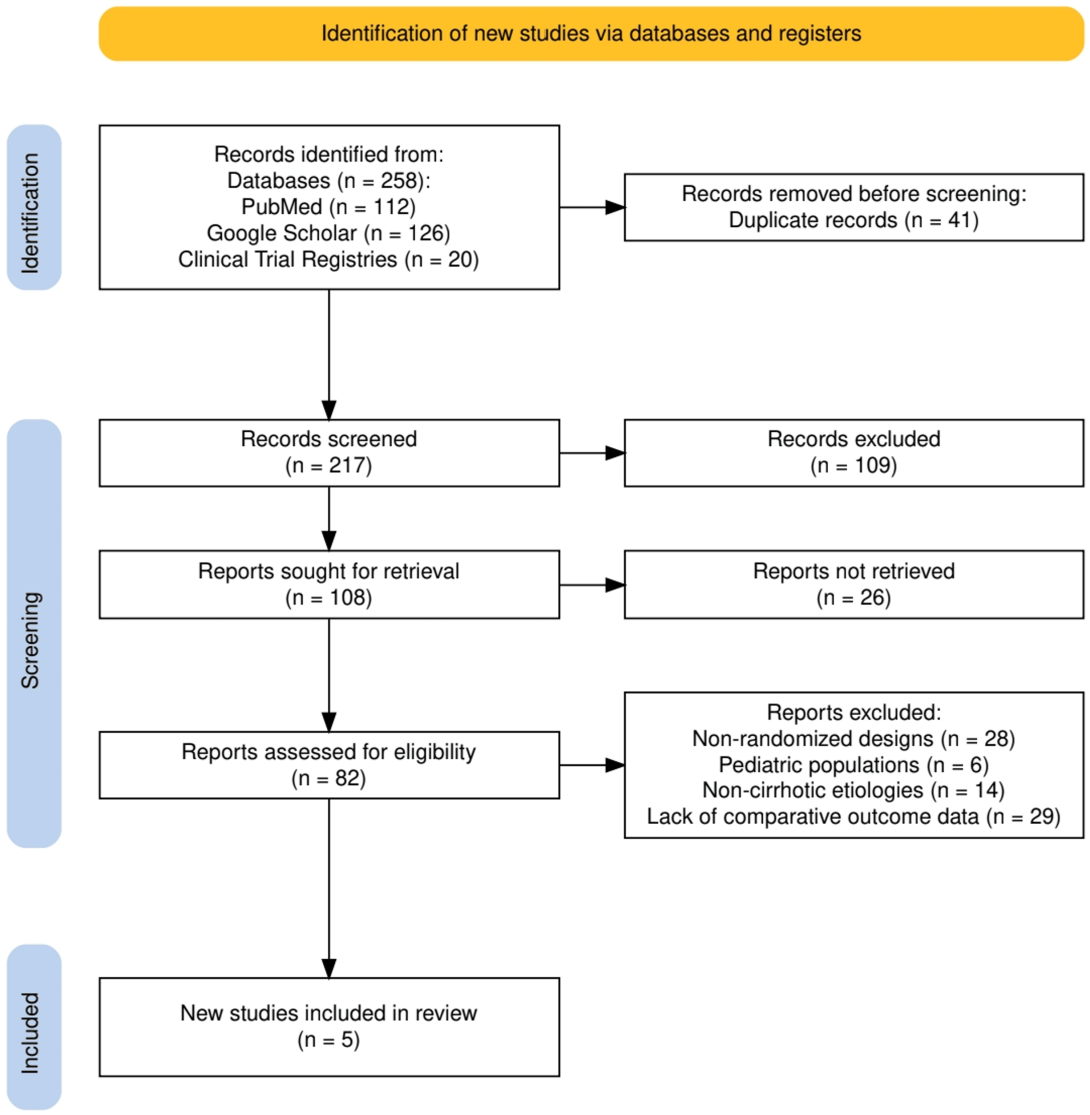
The PRISMA flow chart represents the study selection process. PRISMA: Preferred Reporting Items for Systematic Reviews and Meta-Analyses

A total of 258 records were initially identified through a comprehensive search across three major sources: PubMed (n = 112), Google Scholar (n = 126), and Clinical Trial Registries (n = 20). After removing 41 duplicates, 217 unique records underwent screening. Of these, 109 records were excluded based on title and abstract review. The remaining 108 full-text articles were assessed for eligibility, of which 26 could not be retrieved. The final eligibility assessment included 82 full-text articles, out of which 77 were excluded for reasons including non-randomized designs (n = 28), pediatric populations (n = 6), non-cirrhotic etiologies (n = 14), and lack of comparative outcome data (n = 29). Ultimately, five RCTs met all inclusion criteria and were included in the qualitative synthesis. The entire selection process is summarized in Figure 1, which presents the PRISMA flow diagram for study inclusion.

Characteristics of the Selected Studies

The characteristics of the five selected RCTs are summarized in Table 1. All studies involved adult cirrhotic patients experiencing acute gastroesophageal variceal bleeding, with sample sizes ranging from 42 to 780 participants. Study designs varied from double-blind placebo-controlled trials to open-label and multicenter randomized designs, enhancing the external validity of the findings. The interventions involved standardized dosing of terlipressin - typically beginning with a bolus, followed by maintenance dosing - and were compared against conventional octreotide regimens, including bolus and continuous infusion strategies. The trials evaluated diverse yet clinically significant outcomes, such as bleeding control, mortality, HVPG reduction, blood transfusion requirements, hospital stay duration, and safety profiles. While some studies showed comparable efficacy between terlipressin and octreotide in terms of bleeding control and short-term mortality, others demonstrated distinct advantages with terlipressin in terms of hemodynamic response, durability of HVPG reduction, or improved clinical outcomes in specific subgroups. Collectively, these trials form the evidence base for evaluating the comparative effectiveness of terlipressin and octreotide in acute variceal bleeding.

**Table 1:** The summary of randomized controlled trials comparing terlipressin and octreotide in acute variceal bleeding. RCT: Randomized Controlled Trial; IV: Intravenous; HVPG: Hepatic Venous Pressure Gradient; HR: Heart Rate; MAP: Mean Arterial Pressure; TIPS: Transjugular Intrahepatic Portosystemic Shunt; PVF: Portal Venous Flow

Study (Author, Year)	Study Design	Population (Sample Size)	Intervention (Terlipressin)	Comparator (Octreotide)	Outcomes Assessed	Key Findings
Abid et al., 2009 [10]	Randomized double-blind placebo-controlled trial	Cirrhotic patients with esophageal variceal bleeding (n=324)	2 mg IV bolus, then 1 mg q6h for 72 h	50 µg IV bolus, then 50 µg/h infusion for 72 h	Bleeding control, mortality, blood transfusion, hospital stay, adverse events	Bleeding control: 92.6% (T) vs 95.6% (O); mortality similar; hospital stay shorter in terlipressin group (p ≤ 0.001); no cardiovascular side effects
Li et al., 2021 [11]	Multicenter randomized controlled trial	Decompensated cirrhosis with history of variceal bleeding (n=88)	Administered during HVPG measurement	High-dose octreotide during HVPG measurement	HVPG change, HR, MAP, safety, response	Both reduced HVPG; terlipressin had longer-lasting effect; in alcoholic cirrhosis, 100% response with T vs 0% with O (P=0.005); similar safety profiles
Lv et al., 2019 [12]	Open-label randomized controlled trial	Advanced cirrhosis, acute variceal bleeding (n=129)	Vasoactive drugs + early TIPS within 72 h	Vasoactive drugs + standard care (octreotide)	Transplantation-free survival, mortality, complications	6-week survival: 99% (TIPS) vs 84% (control); 1-year survival: 86% vs 73%; lower mortality in early TIPS group; similar adverse event rates
Seo et al., 2014 [13]	Multicenter randomized non-inferiority trial	Cirrhotic patients with confirmed variceal bleeding (n=780)	Early terlipressin before endoscopy (n=261)	Early octreotide before endoscopy (n=260)	5-day treatment success, bleeding control, rebleeding, mortality	No significant differences: treatment success ~86% (T) vs ~84% (O); similar control, rebleeding, and mortality rates
Baik et al., 2005 [14]	Randomized clinical trial	Cirrhotic patients with prior variceal bleed (n=42)	2 mg IV bolus	100 µg IV bolus + 250 µg/h infusion	HVPG, PVF, MAP, HR	Terlipressin caused sustained HVPG and PVF reductions; octreotide effect was transient and returned to baseline within 5 min; terlipressin had better hemodynamic stability

Quality Assessment

The quality assessment of the included studies, summarized in Table 2, was conducted using the Cochrane RoB 2.0 tool across five domains: randomization process, deviations from intended interventions, missing outcome data, outcome measurement, and selection of reported results. Most studies exhibited a low risk of bias in randomization, outcome measurement, and data completeness. However, a few studies showed some concerns related to reporting bias and small sample sizes, particularly where subgroup analyses were not sufficiently justified or allocation concealment was unclear. One study demonstrated a high risk of bias, primarily due to its open-label design and substantial deviations in intervention protocols between groups, which may have influenced clinical decision-making and outcome interpretation. Despite these limitations, the overall methodological quality of the selected trials was acceptable, allowing for meaningful synthesis and interpretation of clinical outcomes.

**Table 2:** The risk of bias assessment of included trials based on the Cochrane RoB 2.0 tool. RoB: Risk of Bias; RCT: Randomized Controlled Trial; HVPG: Hepatic Venous Pressure Gradient; PVF: Portal Venous Flow; MAP: Mean Arterial Pressure; HR: Heart Rate

Study (Author, Year)	Randomization Process	Deviations from Interventions	Missing Outcome Data	Outcome Measurement	Reported Results	Overall Risk of Bias
Abid et al., 2009 [10]	Low Risk	Low Risk	Low Risk	Low Risk	Low Risk	Low Risk
Li et al., 2021 [11]	Low Risk	Low Risk	Low Risk	Low Risk	Some Concerns (subgroup reporting)	Some Concerns
Lv et al., 2019 [12]	Some Concerns (open-label design)	High Risk (not blinded; treatment arms differ substantially)	Low Risk	Low Risk	Some Concerns	High Risk
Seo et al., 2014 [13]	Low Risk	Low Risk	Low Risk	Low Risk	Low Risk	Low Risk
Baik et al., 2005 [14]	Some Concerns (small sample, unclear allocation concealment)	Low Risk	Low Risk	Low Risk	Some Concerns	Some Concerns

Discussion

This review synthesized data from five RCTs comparing the efficacy of terlipressin and octreotide in cirrhotic patients with esophageal variceal bleeding. Across studies, both agents demonstrated comparable bleeding control and safety outcomes in the general population. For instance, in Abid et al. [10], bleeding control was achieved in 92.6% of patients receiving terlipressin and 95.6% in those on octreotide, with no significant difference in mortality. Similarly, Seo et al. [13] reported five-day treatment success rates of 86.2% for terlipressin and 83.8% for octreotide (p = 0.636), confirming therapeutic equivalence in acute management. However, Li et al. [11] demonstrated that terlipressin provided a more sustained reduction in HVPG, especially in patients with alcoholic cirrhosis, where the response rate reached 100% versus 0% in the octreotide group (p = 0.005). Moreover, Baik et al. [14] showed terlipressin’s sustained effect on portal pressure and flow, contrasting with the transient response seen with octreotide. While overall safety was similar in all trials, Abid et al. [10] noted a significantly shorter hospital stay in the terlipressin group (p ≤ 0.001), suggesting additional economic and logistical benefits. These findings collectively highlight the nuanced but clinically relevant distinctions between the two agents.

Clinically, the sustained hemodynamic effects of terlipressin suggest a potential advantage in maintaining portal pressure control and reducing the risk of rebleeding - a critical goal in the management of variceal hemorrhage [15]. The findings from Li et al. [11] and Baik et al. [14] reinforce this benefit by demonstrating prolonged HVPG suppression with terlipressin, which is mechanistically consistent with its vasopressin-receptor-mediated vasoconstriction in the splanchnic circulation. In contrast, octreotide acts primarily through somatostatin receptor inhibition, leading to mesenteric vasoconstriction but with a shorter half-life and rapid tachyphylaxis [16]. This explains its more transient hemodynamic effects observed across trials. Notably, Li et al. [11] identified a subgroup - patients with alcoholic cirrhosis - that responded significantly better to terlipressin, pointing toward a potential phenotype-specific preference. Furthermore, Lv et al.’s [12] study incorporating early transjugular intrahepatic portosystemic shunts (TIPS) after vasoactive therapy showed markedly better survival (one-year survival: 86% vs. 73%) when terlipressin was part of the pre-TIPS protocol, hinting at synergistic benefits. Therefore, although both agents are guideline-endorsed, terlipressin may be the preferred choice in patients with hemodynamic instability, higher bleeding risk, or alcoholic liver disease, particularly when logistical factors such as hospital stay and long-term bleeding prophylaxis are also considered.

The findings of this review align well with current clinical guidelines, such as those from the American Association for the Study of Liver Diseases (AASLD) [17] and European Association for the Study of the Liver (EASL) [18], both of which recommend terlipressin, somatostatin, or octreotide as first-line pharmacologic agents for acute variceal bleeding. Our analysis corroborates earlier meta-analyses suggesting no significant difference in short-term efficacy among these agents. However, our review expands upon these conclusions by emphasizing the more sustained hemodynamic benefits of terlipressin, particularly in alcoholic cirrhosis [19], as shown by Li et al. [11] and Baik et al. [14]. This nuanced insight is not well captured in broader systematic reviews. By focusing exclusively on recent RCTs and including robust comparative hemodynamic data, our review contributes a more granular understanding of drug-specific advantages that could influence therapeutic decision-making in real-world clinical practice.

From a clinical perspective, the findings suggest that terlipressin may be the preferred agent in settings where sustained portal pressure reduction, prevention of early rebleeding, or shorter hospital stays are prioritized [4]. Its longer half-life and more durable hemodynamic profile may offer superior protection in high-risk patients, including those with alcoholic liver disease or those awaiting TIPS. While octreotide remains an effective and widely available option, it may require more intensive monitoring or adjunctive therapy due to its transient effects [20]. Furthermore, the shorter hospital stay noted with terlipressin [10] (p ≤ 0.001) presents compelling resource allocation advantages in low-resource settings, where cost-effectiveness is critical. These findings can directly inform decisions around drug selection, timing of therapy, and protocol development in both high-income and resource-limited health systems.

This review’s strengths include rigorous inclusion criteria, the exclusive focus on RCTs, and the systematic application of RoB 2.0 assessment, enhancing the reliability of synthesized evidence. Most included studies were recent and conducted across diverse geographic and clinical settings, adding to the generalizability of the findings. The review process adhered to PRISMA guidelines, ensuring transparent reporting and minimizing selection bias. Furthermore, the integration of hemodynamic outcomes (e.g., HVPG and PVF) offers valuable mechanistic insights, not commonly emphasized in prior reviews, which strengthens the clinical applicability of our conclusions.

Despite its strengths, this review has several limitations. First, clinical heterogeneity among the included trials - such as variability in vasoactive regimens, timing of endoscopy, and adjunctive therapies - may impact outcome comparability. For example, Lv et al. [12] used early TIPS, introducing potential confounding. Additionally, open-label designs in some trials could introduce bias in subjective endpoints. Sample sizes were limited in certain studies, such as Baik et al. [14] (n = 42), reducing statistical power. Moreover, the exclusion of non-English publications and unpublished data may have introduced publication bias. While we assessed RoB comprehensively, variations in study quality still affect the overall strength of evidence.

Future research should focus on large-scale, head-to-head RCTs directly comparing terlipressin and octreotide in varied clinical subgroups, such as those stratified by Child-Pugh class or etiology (e.g., alcoholic vs. viral cirrhosis) [21]. The potential role of pharmacogenomics in predicting response [22] to vasoactive agents remains underexplored and could pave the way for personalized therapy [23]. Additionally, studies should evaluate combination strategies, such as optimal timing and sequencing of vasoactive agents with definitive interventions such as TIPS or band ligation. Cost-effectiveness analyses, especially in low-resource settings, are also essential to guide broader implementation of evidence-based protocols.

## Conclusions

This systematic review highlights that, while both terlipressin and octreotide are effective in managing acute gastroesophageal variceal bleeding, terlipressin offers certain clinical advantages, particularly in terms of sustained portal pressure reduction, hemodynamic stability, and potential for shorter hospital stay. These findings are clinically significant, as they support a more tailored approach to drug selection based on patient profile and institutional priorities, especially in settings with limited resources or high disease burden. By synthesizing data from multiple high-quality randomized trials, our work underscores the importance of individualized therapy in cirrhotic patients with variceal hemorrhage, and it reinforces the role of terlipressin as a potentially superior agent in specific subgroups such as those with alcoholic cirrhosis or advanced decompensation. Ultimately, this review contributes meaningful evidence that can inform clinical guidelines, optimize patient outcomes, and support more efficient resource utilization in hepatology care.
